# Compound Event Barrier Coverage in Wireless Sensor Networks under Multi-Constraint Conditions

**DOI:** 10.3390/s17010025

**Published:** 2016-12-24

**Authors:** Yaoming Zhuang, Chengdong Wu, Yunzhou Zhang, Zixi Jia

**Affiliations:** College of Information Science and Engineering, Northeastern University, 110819 Shenyang, China; wuchengdong@ise.neu.edu.cn (C.W.); zhangyunzhou@ise.neu.edu.cn (Y.Z.); jiazixi@ise.neu.edu.cn (Z.J.)

**Keywords:** compound event barrier coverage, multi-constraints, multiplier method, aggregate function, wireless sensor networks

## Abstract

It is important to monitor compound event by barrier coverage issues in wireless sensor networks (WSNs). Compound event barrier coverage (CEBC) is a novel coverage problem. Unlike traditional ones, the data of compound event barrier coverage comes from different types of sensors. It will be subject to multiple constraints under complex conditions in real-world applications. The main objective of this paper is to design an efficient algorithm for complex conditions that can combine the compound event confidence. Moreover, a multiplier method based on an active-set strategy (ASMP) is proposed to optimize the multiple constraints in compound event barrier coverage. The algorithm can calculate the coverage ratio efficiently and allocate the sensor resources reasonably in compound event barrier coverage. The proposed algorithm can simplify complex problems to reduce the computational load of the network and improve the network efficiency. The simulation results demonstrate that the proposed algorithm is more effective and efficient than existing methods, especially in the allocation of sensor resources.

## 1. Introduction

Barrier coverage has been widely used in wireless sensor networks [1]. With the extensive use of WSN applications, different types of sensors, different accuracy requirements and the different costs of sensors represent a severe challenge to network construction [2,3]. This problem is also one of the hot issues in WSNs. In our daily life, barrier coverage has a wide range of applications. For example, barrier coverage can detect illegal immigrants when they are crossing the border in border monitoring applications. Other uses are community security monitoring, battlefield intrusion monitoring and the monitoring of wildlife sanctuaries, etc. [4]. Unlike other coverage issues, barrier coverage pays more attention to detecting targets that cross the barrier area [5].

It is not possible to determine whether there is a target intrusion by relying solely on a single type of sensor data which exceeds a set threshold in a complex monitoring environment. For example, in battlefield monitoring, infrared, sound, vibration and video sensors are all very important monitoring equipment to monitor whether there is an enemy invasion [6]. We cannot simply assume an enemy invasion when the data of a vibration sensor exceeds a threshold, because there may be wind disturbance or large wild animals walking through, so in order to determine the occurrence of an intrusion, we also need to combine the vibration data with data from infrared and video sensors for comprehensive analysis and judgment. The invasion events can be finally detected with high confidence by analyzing the correlations among different types of data. Therefore, the application of multiple types of sensors in the barrier coverage is essential. In this paper, we focus on the barrier coverage problem for a sector in WSNs equipped with different kinds of sensors.

For the current research on barrier coverage, sensors are generally considered to be homogeneous, but in practical applications, many different types and numbers of sensors are often applied in barrier coverage in order to save costs and energy. For instance, a large number of sound, infrared and vibration sensors are applied to assist the video sensors, because sound, infrared and vibration sensors are low cost and have low power consumption, which makes them suitable for long-term continuous work compared to video sensors [7,8].

At the same time, due to the special conditions of the barrier coverage in applications, there will be many constraints to consider, such as time constraints, distance constraints, cost constraints and minimum confidence constraints, etc. Time constraints refer to the fact that the barrier coverage in a battlefield application needs to cover the battlefield area within the stipulated time. Distance constraints mean that in a battlefield or forest fire detection scenario, barrier coverage needs to monitor a specific length of the region, so that the length of the strong barrier coverage area is not less than the target area. Cost constraints mean that in the battlefield monitoring, there are limited material and monetary conditions, to achieve the best coverage effect; the minimum confidence constraints refer to the need for accurate judgment of enemy invasion in battlefield monitoring and the need for accurate monitoring of the occurrence of the forest fire. Specifically, the accuracy of the judgment should not be less than 90%, that is, the minimum confidence constraint is 0.9. There are also some trade-off relations among those constraints. For example, if we require a minimum confidence constraint of 0.90, in order to achieve this confidence, many high confidence sensors such as camera sensors must be used, but the cost of a camera sensor is higher and the sensing radius is also smaller compared to other types of sensors. Due to cost constraints and distance constraints, we cannot use too many camera sensors. These are trade-off relations between the constraints.

In practical application, after the sensor resource allocation, the sensors will undergo lots of problems while working, such as stopping working, information transmission errors and so on. In order to solve the above problems, according to the monitoring performance and stability of different sensors, the sensors are assigned different confidences. These confidences reflect the accuracy and stability of the sensors. Therefore, it is necessary to propose a series of systems to study the compound event barrier coverage in wireless sensor networks, so that the monitoring performance of the barrier coverage to achieve the best performance when the network is constrained by all the above constraints.

The event barrier coverage is a novel coverage problem which is different from the traditional barrier coverage where deployment costs of sensors, total budget and compound events are not considered. The traditional barrier coverage is concerned about whether there is a target intrusion. According to the monitoring requirements, it can also be classified as strong barrier coverage and weak barrier coverage, while event barrier coverage is concerned with the cost of sensors, and the total budget for the monitoring process. The goal of the barrier coverage is to monitor target intrusion, while the purpose of the event barrier coverage is to study how to monitor events more effectively under multiple constraints.

A compound event is constituted by the sub-events which satisfy specific temporal and spatial constraints in a barrier area. The occurrence of a compound event indicates that all the corresponding sub-events occur. However, the occurrence of corresponding sub-events cannot guarantee that a compound event occurs. The accumulation of the confidence of individual sub-events can be considered as the sign of the occurrence of a compound event in a barrier area. To the best of our knowledge, this is the first work to study the event barrier coverage problem.

There have been a series of works on event problems. Gao et al. [9] first proposed the event detection problem in heterogeneous sensor networks. On the basis of event detection, the problem of compound event coverage under a single cost constraint has been considered [10]. The above study only considered the ideal application scenario. However, in many practical applications, people tend to pay more attention to monitoring the performance after deployment, rather than just the initial coverage conditions.

With the development of science and technology, sensors have become cheaper and cheaper, but it is still practically impossible to deploy a large number of the same sensors over a long border in practical application, such as battlefield monitoring. For example, China has 22,000 km of border, so if a video sensor has a sensing range of 100 m, and the cost of each video sensor node is $10, the cost for full border coverage would be enormous. On the other hand, using low-precision vibration or sound sensors, etc., the monitoring performance cannot meet the needs. The barrier coverage is often used to monitor narrow strip areas, so it is important to investigate the use of various types of sensors to complement each other for monitoring performance.

Traditional combinatorial optimization methods, such as the genetic algorithm, simulated annealing algorithm and particle swarm algorithm [11,12,13,14,15], cannot be applied to compound event barrier coverage. First, the different sub-events are not independent of each other. For example, in river monitoring, if a river is contaminated, the toxins and pH in the river will change. At the same time, due to the death of microorganisms in the water, the temperature of the river will rise. The elevated temperature in the water will cause further changes in toxins and pH. Accumulation of different sub-events will lead to the occurrence of the target event. At the same time, some sensors are cheap, but important; while other sensors are expensive but not so important in the construction of barrier coverage which does not meet our usual knowledge. Second, according to different monitoring requirements, we require the premise of not reducing other performance, so that a performance can achieve an optimal, rather than a global optimal value. For example, the shortest deployment time, or the lowest cost, or the best coverage quality, and so on. Finally, due to the limitations of multiple constraints, the traditional combinatorial optimization methods will require a large amount of computation which cannot meet the requirements of real-time systems.

Based on the above analysis, compound event barrier coverage can effectively solve the joint application problem of a variety of sensors in barrier coverage. Our contributions may be summarized as follows:

- The compound event barrier coverage problem is formulated based on a joint probability model. At the same time, the joint probabilistic model is used to solve the problem of effectively merging sub-event confidence in the barrier coverage problem. To the best of our knowledge, this is the first work to study the compound event barrier coverage optimization problem.
- The problem of compound event barrier coverage with time constraints, distance constraints, cost constraints and minimum confidence constraints is proposed. In battlefield applications, in order to take the preemptive actions, it is necessary to complete the barrier coverage within a limited time, so the barrier coverage problem is time-bound. At the same time, due to the complex terrain of the battlefield, such as the existence of rivers and minefields, the barrier coverage path will be limited. In battlefields and other hazardous environment, the logistics supply will be limited, so the barrier coverage will also be subject to cost constraints. In this paper, a multiplier method based on active-set strategy is proposed, which effectively solves the problem of compound event barrier coverage under time constraints, distance constraints, cost constraints and minimum confidence constraints, etc. To the best of our knowledge, this is the first work to study the compound event barrier coverage optimization problem under multiple constraints.
- The effectiveness and efficiency of our compound event barrier coverage mechanism are better than previous algorithms as proved by extensive simulations. The results show that our technique is more computationally efficient, especially when the network topology is relatively complex.

The rest of the paper is organized as follows: Section 2 briefly introduces related work. In Section 3, we present the definitions of the compound events model based on joint probability density. In Section 4, we analyze the problem of compound event barrier coverage optimization, and propose a multiplier method based on active-set strategy to solve the problem of compound event barrier coverage optimization under multiple constraints. The simulation experiments and evaluation are given in Section 5, and compared with the latest event coverage algorithm. Finally, the conclusions and prospects for future work are offered in Section 6.

## 2. Related Works

In order to effectively monitor the target area, it is necessary to deploy sensors according to the network monitoring task [16,17,18]. The paper [19] is the first to consider target coverage and connectivity jointly for WHSNs with multiple sensing units in heterogeneous wireless sensor networks (HWSNs). Wireless communication between sensors allows the formation of flexible sensor networks, which can be deployed rapidly over wide or inaccessible areas [20]. In [21], the authors proposed an adaptation of the gradient descent method to optimize the position and orientation of sensors to solve the sensor placement problem. The novelty of the proposed method lies in the combination of gradient descent optimization with a realistic model, which considers both the topography of the environment and a set of sensors with directional probabilistic sensing. The paper [22] proposes a depth-adjustment deployment algorithm based on two-dimensional convex hull and spanning tree for UWSNs. In mobile wireless sensor networks, nodes are allowed to move autonomously for deployment. The paper [23] presents an experimental evaluation of both reactive deployment approaches: rule-based and behavior-based ones which tend to provide better coverage and communication balance, especially for a large number of nodes in areas with obstacles.

Coverage problems are generally divided into three categories: point coverage, area coverage [24] and barrier coverage [25]. Barrier coverage is primarily used to monitor targets which cross the target area [26]. Barrier coverage can also be divided into strong barrier coverage, weak barrier coverage, k-barrier coverage and so on. The research of compound event barrier coverage is a new research field, which mainly focuses on the reasonable deployment of sensor resources in barrier coverage, in order to achieve the optimization of coverage quality [10]. The paper [27] constructs a directional barrier graph to provide strong barrier coverage over a given belt region. In [28], the authors believe that sometimes detecting intruders is not sufficient, so a strong k-barrier coverage algorithm is proposed to detect an intruder and distinguish whether the intruder is legal or not.

With the gradual deepening of research in barrier coverage, there is also a growing requirement for monitoring, so many types of sensors are required to complete the barrier coverage together [29]. Different types of sensors have different properties and coverage performance. The paper [30] proposes a centralized connected target k-coverage algorithm to solve coverage problems by coordinating relations between heterogeneous sensors. By using heterogeneous sensors, a novel greedy barrier construction algorithm is proposed to solve the problem that one barrier is not robust to provide barrier coverage under both sunny and rainy weather [31]. However, the above discussion simply uses the characteristics of heterogeneous sensors which does not take into account the confidence merging problem between the sensors and sensor resource allocation problem based on monitoring events. With the increase of monitoring requirements, there is an urgent need to study event-based barrier coverage problems.

An approximate compound event detection problem is investigated for the first time where compound events are integrated by multi-mode data generated by different types of sensors. Algorithms are proposed to compute the optimal transmitting scheme with minimum cost as the constraint when the confidence of the compound event exceeds the threshold [9]. On the basis of event detection, the problem of compound event coverage under single cost constraint is considered in [10]. The authors believe that the occurrence of a compound event is the cumulative result of multiple sub-events. A wireless sensor network is designed to detect single (or atomic) events or compound events and ensure the coverage and connectivity conditions [32]. The above work assumes that wireless sensor networks work in an ideal environment [33,34,35]. However, in practical applications, the barrier coverage works in complex environments, and compound event barrier coverage is subject to many constraints. So far, no studies have been done on compound event barrier coverage under multi-constraints conditions.

For multi-constrained optimization problems, Pareto frontier optimization methods and multiplier methods are usually employed. In [36], the authors adopted a multi-objective particle swarm optimization based on Pareto optimality to solve such multi-objective optimization problem and find a better tradeoff between time delays and energy consumption. In [37], the authors take advantage of the Pareto-optimal theory and technique to select appropriate cluster heads in wireless sensor networks. A large number of sensor nodes in the network will produce a huge computational load. Considering the complexity of the Pareto front algorithm, the multiplier method will be more suitable for sensor network applications, so in [38], the authors make use of consensus optimization in conjunction with the alternating direction method of multipliers (ADMM). This proposed algorithm converges significantly faster as compared to the traditional methods.

Studies on deployment strategy, barrier coverage, heterogeneous sensor networks, event coverage and multi-constrained optimization problems are discussed, respectively. The paper [10] cannot propose an effective method for calculating merging confidence of compound event coverage in wireless sensor networks. At the same time, this paper only studies the problem of event coverage under a single cost constraint which cannot satisfy the application requirements in complex environments because in practical applications, the barrier coverage problem will be subject to a variety of constraints, like time constraints, distance constraints, cost constraints and minimum confidence constraints and so on, so it can be seen that there are some gaps in the study of compound event barrier coverage under multiple constraints. Therefore, this paper presents a compound event barrier coverage model with multiple constraints, which can calculate the merging confidence of compound event effectively and distribute the sensor network resource reasonably.

## 3. The Event Model

The existing event coverage model is a compound event model proposed by Gao et al. [9]. An event in barrier coverage is defined as an occurrence of an invasion event or an object during a period of time through a barrier area. Events are classified into sub-events and compound events. A sub-event characterizes a state of the physical world or the information of an object reflected by a single sensing value, e.g., a numerical value exceeding some given threshold, or a meaningful pattern in an audio stream. A compound event is composed by atomic events satisfying multi-constraint conditions, such as temporal and spatial constraints, which expresses an observable occurrence of a complex phenomenon or an object. The sub-events satisfying multi-constraints, such as temporal constraints or spatial constraints can constitute a compound event. The occurrence of a compound event indicates that all the corresponding sub-events occur. For example, in a forest fire monitoring system, temperature sensors, infrared sensors, smoke density sensors and camera sensors are deployed in the monitored field to monitor the occurrence of the forest fire. When the forest fire occurs in the monitored area, anomalies can be detected by the four types of sensors and the corresponding data are generated. By jointly processing all these data, a “forest fire occurrence” event can be determined.

Therefore a sub-event is defined as an intrusion target that triggers a single sensor in the barrier area. For example, targets that invade the barrier area can raise the environment temperature, which triggers an infrared sensor. More specifically, an sub event is defined by *s*(*t*, *c*, *E*), where *t* is the time of the event occurrence, which could be a punctual time point or a time-interval, *c* is the coordinate where the event occurs, which could be either a point or a track, and *E* is used to define the threshold value of the event occurring, represented by a logic expression. For example, sub-event *e*(*t*, *c*, *E*) = (15/8/2016, (*x*, *y*), *Vibration* > 5 mm/s), expresses the vibration at location (*x*, *y*) on 15 August 2016 is greater than 5 mm/s.

Compared with the traditional area coverage, the barrier coverage does not need to cover the entire area, but only needs to ensure the effective monitoring of targets which pass through the sensor network. The barrier coverage pays more attention to the concept of Target of Interest (TOI). For example, in forest fire monitoring, firefighters are more concerned about the coverage quality of the fire area to forecast the spread of a fire. In battlefield monitoring, the army is more concerned about the coverage quality in the front of the battlefield region, to determine whether there is enemy invasion. In environmental applications, quality inspectors are more concerned about the coverage quality in particular waters to determine whether there is a pollution situation.

Therefore, in the event barrier coverage, depending on the monitoring needs of different scenarios, we focus on monitoring values of specific sensors in specific areas, that is, the probability of the sub-event. For example, in forest fire monitoring, event barrier coverage focuses on the monitoring of temperature sensors in the fire area, namely, the occurrence probability of the sub-event that temperature greater than the threshold. The confidence of the occurrence of the compound event can be obtained by analyzing the confidence of the sub-events. Therefore, how to calculate the confidence of the compound event reasonably has become a very important issue.

In barrier coverage, the confidence of a compound event is combined by the confidence of many sub-events. The compound event is considered to occur when the confidence level of the compound event reaches the threshold value. The traditional method [10] only defines a combination operator, and does not propose an exact calculation method. In this paper, the joint probabilities method can be used to calculate the merged confidence of the sub-events effectively.

The coverage quality of coverage mechanism α = {α_1_, α_3_} represents the combination of confidence of sub events generated by the nodes from category 1 and 3, namely, *f*(α) = *f*(α_1_Θα_3_) = *g*(α_1_Θα_3_). Also, the coverage quality of α = {α_1_, α_2_, α_3_, α_4_} is *f*(α) = *f*(α_1_Θα_2_Θα_3_Θα_4_) = *g*(α_1_Θα_2_Θα_3_Θα_4_), where α is the confidence of the different types of sub-events, that is, the confidence of different sensors; *f*(α) is the compound event; *f*(α) is defined by α_1_Θα_2_Θα_3_Θα_4_.

**Definition** **1 (Joint Probability).** *p_i_ is the confidence of sub-event* α*_i_. P is the confidence of the compound event after merging. The combination operator Θ is defined as calculating the joint probability*.

*Therefore, the confidence formula can be obtained:*

(1) $$P=1-\prod_{i=1}^{n}\left(1-p_{i}\right)$$

*From the nature of the joint probability method:*

(2) $$P\geq MIN(p_{i})$$

For example, *e*_1_(*t*, *c*, *E*) = (*t*_1_, (*x*_1_, *y*_1_), *toxin level* > 5 g/mL), represents the event of the toxin level at time *t*_1_ in location (*x*_1_, *y*_1_) being greater than 5 g/mL.

*e*_2_(*t*, *c*, *E*) = (*t*_2_, (*x*_2_, *y*_2_), *pH* > 7.0) represents the event of the pH value at *t*_2_ in location (*x*_2_, *y*_2_) being greater than 7.0. The compound event which indicates a pollution breakout can be speculated by the sub events *e*_1_ and *e*_2_, i.e., *E*[(*e*_1_, 0.3), (*e*_2_, 0.6), *t*_1_, *s*_1_] = (*toxin level* > 5 g/mL ∩ *pH* > 7.0 ∩ *t*_1_ = *t*_2_ ∩ *s*_1_ = *s*_2_) = 0.72. This means that the probability of the pollution breaking out is 0.72, if the two sub-events happen in the same location and at the same time. This is the first work to calculate the confidence of compound event coverage accurately, while previous works were based on historical data and the experience of professionals [10]. For simplicity, the arithmetic operator “*” is adopted as the symbol of the joint probability in the following content.

## 4. Compound Event Barrier Coverage Optimization Problem

### 4.1. Main Idea

The objective of the study of compound event barrier coverage optimization is to allocate each type of sensors to optimize the barrier coverage so as to achieve better performance under multi-constraint conditions.

Within a barrier coverage area, a compound event is monitored by *n* different types of sensors which constitute each sub-event. The occurrence of a compound event *E* is the result of the accumulation of several sub-events *e*. If there are no time, distance and cost constraints, we can increase the number of sensors to get better monitoring performance in barrier coverage. However, unlike traditional coverage scenarios, barrier coverage is subject to many constraints due to its application in hazardous and complex environments.

### 4.2. Problem Formulation

The following optimization problems with various multi-constraints are established:

(3) $$\begin{array}{l} \text{max }f(\alpha )=f(\alpha_{1},\alpha_{2},\ldots ,\alpha_{n})\\ s.t.\;f^{i}(\alpha )=g_{i}(\alpha_{1},\alpha_{2},\ldots ,\alpha_{n})\leq 0(i=1,2,\ldots ,m) \end{array}$$

where *f*(α) is the coverage optimization function. α represents different types of sensors. *f^i^*(α) represents multi-constraints. *g_i_*(α) is a constraint function. Solving the variable vector $\alpha ={\left[\alpha_{1},\alpha_{2},\ldots ,\alpha_{n}\right]}^{T}(x\in R^{n})$, which satisfied *m* constraints so that the objective function *f*(α) is maximized, that is, the coverage ratio is the maximum.

For Equation (3), the relaxation factor *y_i_* is introduced into the equality constrained optimization problem:

(4) $$\begin{array}{l} \text{max }f^{0}(\alpha ),\\ s.t.\;f^{i}(\alpha )+y_{i}=0\;(i=1,2,\ldots ,m) \end{array}$$

Construct an augmented objective function:

(5) $$\tilde{\psi }(\alpha ,y,\lambda ,\sigma )=f^{0}(\alpha )-\sum_{i=1}^{m}\lambda_{i}[f^{i}(\alpha )+y_{i}^{2}]+\frac{\sigma }{2}\sum_{i=1}^{m}{\left[f^{i}(\alpha )+y_{i}^{2}\right]}^{2}$$

In order to optimize the model and reduce the computational complexity of the model, the problem is transformed:

(6) $$\begin{array}{l} \text{max }f^{0}(\alpha ),\\ s.t.\;f_{\text{max}}(\alpha )=\text{max}\{f^{i}(\alpha )\}\leq 0. \end{array}$$

The feasible region is Ω. Therefore, when constructing an augmented Lagrange function, we only need to introduce a multiplier *λ* ∈ *R* and an auxiliary variable *y* ∈ *R*. Although the current maximal function cannot inherit the smoothness of the original function, it can approximate the maximum function using the aggregation function. The aggregation function is also called exponential penalty function which can be derived from the maximum entropy principle [39]. Aggregate function can be expressed as:

(7) $$f_{p}(\alpha )=\frac{1}{p}\text{ln}\left(\sum_{i=1}^{m}\text{exp}\{pf^{i}(\alpha )\}\right),$$

where *p* is the smoothing parameter, *p* > 0.

**Definition** **2 (Monotonicity).** *f_p_*(α) *decreases monotonically with the increase of p. At the same time, there is:*

(8) $$f_{\text{max}}(\alpha )\leq f_{p}(\alpha )\leq f_{\text{max}}(\alpha )+\frac{1}{p}\text{ln }m$$

*We can get the following relation by calculating f_p_(α) gradients and Hesse arrays:*

(9) $$\nabla f_{p}(\alpha )=\sum_{i=1}^{m}\mu_{i}(\alpha ,p)\nabla_{\alpha }f^{i}(\alpha ),$$

(10) $$\begin{array}{c} \nabla_{\alpha \alpha }f_{p}(\alpha )=\sum_{i=1}^{m}\mu_{i}(\alpha ,p)\nabla_{\alpha \alpha }f^{i}(\alpha )+p\sum_{i=1}^{m}\mu_{i}(\alpha ,p)\nabla_{\alpha }f^{i}(\alpha )\nabla_{\alpha }f^{i}{\left(\alpha \right)}^{Τ}-\\ p\sum_{i=1}^{m}\mu_{i}(\alpha ,p)\nabla_{\alpha }f^{i}(\alpha )\sum_{i=1}^{m}\mu_{i}(\alpha ,p)\nabla_{\alpha }f^{i}{\left(\alpha \right)}^{Τ} \end{array}$$

*In Equation (10):*

(11) $$\mu_{i}(\alpha ,p)=\frac{\text{exp}\{pf^{i}(\alpha )\}}{\sum_{i=1}^{m}\text{exp}\{pf^{i}(\alpha )\}}\in (0,1]$$

(12) $$\sum_{i=1}^{m}\mu_{i}(\alpha ,p)=1$$

*In order to reduce the computational cost of aggregate functions, an active-set strategy is used [40]. For the aggregation function f_p_(α), the subset* Ω⊂M *is considered. Define the function* $f^{\Omega }:R^{n}\to R$ *as:*

(13) $$f^{\Omega }(\alpha )=\underset{i\in \Omega }{\text{max}}\{f^{i}(\alpha )\}$$

*The smooth approximation function is:*

(14) $$\begin{array}{ll} f_{p}^{\Omega }(\alpha ) & =\frac{1}{p}\text{ln}(\sum_{i\in \Omega }\text{exp}\{pf^{i}(\alpha )\})\\ & =f^{\Omega }(\alpha )+\frac{1}{p}\text{ln}(\sum_{i\in \Omega }\text{exp}\{pf^{i}(\alpha )-f^{\Omega }(\alpha )\}) \end{array}$$

*We can get:*

(15) $$\nabla_{\alpha }f_{p}^{\Omega }(\alpha )=\sum_{i\in \Omega }\mu_{i}(\alpha ,p)\nabla_{\alpha }f^{i}(\alpha ),$$

*In Equation (15):*

(16) $$\mu_{i}(\alpha ,p)=\frac{\text{exp}\{pf^{i}(\alpha )\}}{\sum_{k\in \Omega }\text{exp}\{pf^{k}(\alpha )\}}\in (0,1].$$

**Definition** **3 (Active-set Strategy).** *For any of* $\alpha \in R^{n}$ *and* i∈Ω*, the following equation is satisfied:*

(17) $$\underset{p\to \infty }{\text{lim}}\mu_{i}(\alpha ,p)=\left\{ \begin{array}{ll} \frac{1}{\left|\Omega (\alpha )\right|} & ,i\in \hat{\Omega }(\alpha )\\ 0 & ,Otherwise \end{array}\right.$$

(18) $$\underset{p\to \infty }{\text{lim}}p\mu_{i}(\alpha ,p)=0,i\notin \hat{\Omega }(\alpha )$$

(19) $$\underset{p\to \infty }{\text{lim}}\nabla_{\alpha }f_{p}^{\Omega }(\alpha )=\sum_{i\in \Omega (\alpha )}\frac{1}{\left|\Omega (\alpha )\right|}\nabla_{\alpha }f^{i}(\alpha )$$

*where* Ω(α) *represents:*

(20) $$\Omega (\alpha )=\{i\in M|f(\alpha )=f^{\Omega }(\alpha )\}$$

The study of [40] shows that the active-set strategy can reduce the gradient calculation by 75%.

*Equation (6) is simplified:*

(21) $$\begin{array}{l} \text{max }f^{0}(\alpha ),\\ s.t.\;f_{p}(\alpha )\leq 0. \end{array}$$

*The relaxation factor* y∈R *was introduced for transformation:*

(22) $$\begin{array}{l} \text{max }f^{0}(\alpha ),\\ s.t.\;f_{p}(\alpha )+y^{2}\leq 0. \end{array}$$

*Then the augmented Lagrange function is:*

(23) $$\psi_{p}(\alpha ,y,\lambda ,\sigma )=f^{0}(\alpha )-\lambda [f_{p}(\alpha )+y^{2}]+\frac{\sigma }{2}{\left[f_{p}(\alpha )+y^{2}\right]}^{2}$$

*In order to eliminate y,* ${\tilde{\psi }}_{p}(\alpha ,y,\lambda ,\sigma )$ *is minimized with respect to y by letting* ${\tilde{\psi }}_{p}(\alpha ,y,\lambda ,\sigma )=0$*, so we can get:*

(24) $$y[\sigma y^{2}-(\lambda -\sigma f_{p}(\alpha ))]=0$$

*Then:*

(25) $$y^{2}=\left\{ \begin{array}{ll} \frac{1}{\sigma }[\lambda -\sigma f_{p}(\alpha )] & ,\lambda -\sigma f_{p}(\alpha )＞0\\ 0 & ,Otherwise \end{array}\right.$$

*Thus:*

(26) $$-\lambda [f_{p}(\alpha )+y^{2}]+\frac{\sigma }{2}{\left[f_{p}(\alpha )+y^{2}\right]}^{2}=\frac{1}{2\sigma }[{\left(\text{min}\{0,\lambda -\sigma f_{p}(\alpha )\}\right)}^{2}-\lambda^{2}]$$

*Equation (26) was taken back into* ${\tilde{\psi }}_{p}(\alpha ,y,\lambda ,\sigma )$ *to eliminate y. We can then get:*

(27) $${\tilde{\psi }}_{p}(\alpha ,\lambda ,\sigma )=\underset{y}{\text{min}}{\tilde{\Psi }}_{p}(\alpha ,y,\lambda ,\sigma )=f^{0}(\alpha )+\frac{1}{2\sigma }[{\left(\text{min}\{0,\lambda -\sigma f_{p}(\alpha )\}\right)}^{2}-\lambda^{2}]$$

*Equation (25) is brought into the multiplier iteration formula* $\lambda_{k+1}=\lambda_{k}-\sigma (f_{p}(\alpha^{k})+y^{2})$*. Then:*

(28) $$\lambda_{k+1}=\left\{ \begin{array}{ll} 0 & ,\lambda_{k}-\sigma f_{p}(\alpha^{k})＞0\\ \lambda_{k}-\sigma f_{p}(\alpha^{k}) & ,Otherwise \end{array}\right.$$

*After simplification:*

(29) $$\lambda_{k+1}=\text{min}\{0,\lambda_{k}-\sigma f_{p}(\alpha^{k})\}$$

*Similarly, Equation (25) is brought into* $|f_{p}(\alpha^{k})+y_{k}^{2}|\leq \varepsilon$*. Then we can get the terminate condition:*

(30) $$\text{min}\{|f_{p}(\alpha^{k})|,|\frac{\lambda_{k}}{\sigma }|\}\geq \varepsilon$$

### 4.3. Active Set Multiplier Policy (ASMP)

Through the analysis of the model, it is converted to solve the following equation.

(31) $$\underset{y}{\text{min}}{\tilde{\Psi }}_{p}(\alpha ,\lambda ,\sigma )=f^{0}(\alpha )+\frac{1}{2\sigma }[{\left(\text{min}\{0,\lambda -\sigma f_{p}(\alpha )\}\right)}^{2}-\lambda^{2}]$$

Then a multiplier method based on active-set strategy is designed to solve above problem. Because this algorithm is designed for the fixed parameters $p_{i},\lambda_{i},\sigma_{i}$, for the sake of convenience, we denote $\tilde{\psi }(\alpha )={\tilde{\psi }}_{p_{i}}(\alpha ,\lambda_{i},\sigma_{i})$ and ${\tilde{\psi }}^{\Omega }(\alpha )={\tilde{\psi }}^{\Omega }(\alpha ,\lambda_{i},\sigma_{i})$. At the same time, for ∀ε≥0, we denote set-valued mapping:

(32) $$Q_{\varepsilon }：R^{n}\to 2^{M},Q_{\varepsilon }(x)=\{j\in M|f_{\text{max}}(\alpha )-f^{j}(\alpha )\leq \varepsilon \}.$$

The details of the procedure for solving sub-events are given in Algorithm 1.

**Algorithm 1:** Sub-event Algorithm1: Initialization: $t=0,\alpha^{i0}=\alpha^{i-1},\alpha \in (0,\frac{1}{2}),\beta \in (0,1),\bar{\varepsilon }>0,\Omega_{0}=\Omega_{t}(\alpha^{i0}),B_{0}=E(\text{Unit Array}).$2: Solve the equation set $B_{t}d=-\nabla_{x}{\tilde{\psi }}^{\Omega_{t}}(\alpha^{it})$ for d. Determine the search direction $d^{t}$.3: Determine the smallest non-negative integer for $m_{t}$ that satisfies the constraint: ${\tilde{\psi }}^{\Omega_{t}}(\alpha^{it}+\beta^{m_{t}}d^{t})-{\tilde{\psi }}^{\Omega_{t}}(\alpha^{it})\leq \alpha \beta^{m_{t}}\nabla_{\alpha }{\tilde{\psi }}^{\Omega_{t}}(\alpha^{it})d^{t}$4: Do $\tau_{t}=\sigma^{m_{t}},\alpha^{i,t+1}=\alpha^{it}+\tau_{t}d^{t}$, $\Omega_{t+1}=\Omega_{t}\cup Q_{\varepsilon }(\alpha^{i,t+1})$;5: Judge, if $\left\|\nabla_{\alpha }{\tilde{\psi }}^{\Omega_{t+1}}(\alpha^{i,t+1})\right\|\leq \bar{\varepsilon }\;d^{t}$, make $\alpha^{i}=\alpha^{i,t+1}$, then exit; Otherwise, set t=t+1, Skip back to step 1.

Considering the property of each sub-event, as given in the above equations, the procedure for solving each compound event is given in Algorithm 2.

**Algorithm 2:** Active Set Multiplier Policy (ASMP)1: Initialization: $\alpha^{0}\in R^{n},p_{1}＞0,\hat{p}＞0,\lambda_{1}\in R,\sigma_{1}＞0,\mu ＞1,\mu ＞1,\varepsilon (p)＞0.$2: Set i=1.3: Solving sub-events: Utilizing $\alpha^{i-1}$ as the initial point, use Algorithm 1 to solve the unconstrained problem $\text{max}{\tilde{\Psi }}_{p_{i}}(\alpha ,\lambda_{i},\sigma_{i})$ to get the maximum point $\alpha^{i}$;4: Verify termination conditions: If $\left\|\nabla_{\alpha }{\tilde{\psi }}_{p_{i}}(\alpha^{i},\lambda_{i},\sigma_{i})\right\|\leq \varepsilon (p_{i})$ does not satisfied, skip to step 3; if $p_{i}＞\hat{p}$, then stop; otherwise set $p_{i+1}=\mu p_{i},\alpha^{i+1}=\alpha^{i},i=i+1$, go to step 2;5: Update penalty parameters: If $\left\|\nabla_{\alpha }{\tilde{\psi }}_{p_{i}}(\alpha^{i},\lambda_{i},\sigma_{i})\right\|\geq \nabla_{\alpha }{\tilde{\psi }}_{p_{i}}(\alpha^{i-1},\lambda_{i},\sigma_{i})\|$, set $\sigma_{i+1}=\mu \sigma_{i}$; otherwise set $\sigma_{i+1}=\sigma_{i}$;6: Update multipliers: $\lambda_{i+1}=\text{min}\{0,\lambda_{i}-\sigma_{i}f_{p_{i}}(\alpha^{i})\}$;7: Set $\lambda_{i+1}=\text{min}\{0,\lambda_{i}-\sigma_{i}f_{p_{i}}(\alpha^{i})\}$, skip to step 1.

**Notation** **1.** *For the standard BFGS correction, the use of Armijo search cannot guarantee the iterative matrix of positive definite. Therefore, in step 1 of Algorithm 1, the modified BFGS correction formula is used for $B_{t}$:*

(33) $$B_{t}=B_{t-1}-\frac{B_{t-1}s_{t-1}s_{t-1}^{T}B_{t-1}}{s_{t-1}^{T}B_{t-1}s_{t-1}}+\frac{z_{t-1}z_{t-1}^{T}}{s_{t-1}^{T}z_{t-1}}$$

*In the formula:*

$$s_{t-1}=\alpha^{it}-\alpha^{i,t-1};z_{t-1}=\theta_{t-1}{\bar{y}}_{t-1}+(1-\theta_{t-1})B_{t-1}s_{t-1};{\bar{y}}_{t-1}=\nabla_{\alpha }{\tilde{\psi }}^{\Omega_{t}}(\alpha^{it})-\nabla_{\alpha }{\tilde{\psi }}^{\Omega_{t-1}}(\alpha^{i,t-1}).$$

*where:*

(34) $$\theta_{t-1}=\left\{ \begin{array}{ll} 1 & ,s_{t-1}^{T}{\bar{y}}_{t-1}\geq 0.2s_{t-1}^{T}B_{t-1}s_{t-1}\\ \frac{0.8s_{t-1}^{T}B_{t-1}s_{t-1}}{s_{t-1}^{T}B_{t-1}s_{t-1}-s_{t-1}^{T}{\bar{y}}_{t-1}} & ,Otherwise \end{array}\right.$$

By using a multiplier method based on active-set strategy, the problem of event coverage under multi-constraint conditions can be solved effectively. The multiplier method based on an active-set strategy (ASMP) is used to solve the trade-offs among constraints by transforming the multiple constraints into multi-inequality constraints. In the multiplier method, the multi-inequality constraints represent the multiple constraints; the variable to be solved is the number of each sensor. In the process of optimization with the multiplier method, the objective function optimizes the coverage quality of the network, and the number of each sensor and the final coverage ratio are obtained. Then simulation experiments are used to prove it.

## 5. Experiments and Evaluation

In this section, we evaluate the performance of the proposed algorithms through simulations and compare our results with the OCQ-Max-fit, OCQ-Greedy and OCQ-Naïve algorithms using the same experimental parameters.

### 5.1. Environment Settings

We use MATLAB2014a to perform the ASMP simulation experiments. In the experiments, we use five types of sensors. The confidence of the five different types of sensors is 0.05, 0.1, 0.45, 0.15 and 0.25, respectively. Each type of sensors has different deployment time, perceived radius, and deployment cost, depending on its nature. The parameter meanings for our experiments are presented in Table 1. The monitoring area is set at a band width of 600 m × 100 m. Experiments are conducted five times with the aim of covering at each condition for evaluation purposes, and these experiments are performed on a desktop computer equipped with an Intel(R) Core(TM) i5-4590 CPU @ 3.30 GHz, an 4-GB memory and the 64-bit Windows 7 operating system.

### 5.2. Experimental Evaluation

Experiments have been conducted for evaluating the performance and efficiency of our compound event barrier coverage mechanisms under multi-constraints. The time constraints, distance constraints, cost constraints and minimum confidence constraints for four cases are presented in Table 2. The results of our experimental evaluation are presented and discussed in the following.

**Figure 1:**
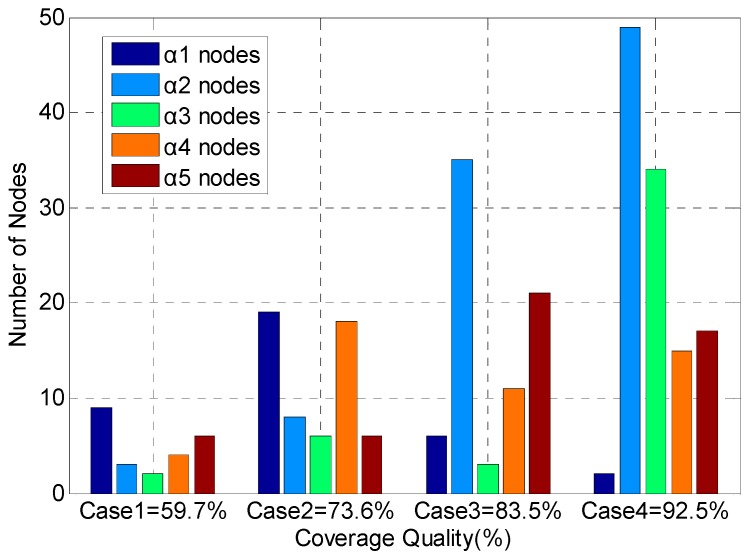
Sensor resource allocation for the compound event barrier coverage under multi-constraint conditions. As the number of nodes increases, the network coverage gradually increases.

In Figure 1, Case 1 has a harsh time constraint, so more optical density sensors which can be deployed relatively fast have been used. Case 2 has a strict distance constraint, so the optical density sensors and smoke density sensors which have larger perceived radius have been more used. Case 3 has a limited cost constraint, so the number of the lower cost temperature sensors is 35. This number is relatively large. At the same time, taking into account the need to meet the requirement of high confidence, the number of infrared sensors is also 34. Case 4 has a strict minimum confidence requirement, so the video sensors which have higher confidence have been used in a large number of applications, compared to the former three conditions. However, under the cost and deployment time constraint, the number of video sensors is limited to 34. The number of temperature sensors (49) is the largest. The simulation results show that the ASMP algorithm can allocate sensor resources effectively under multiple constraint conditions, and make the performance of barrier coverage meet the requirements of the application.

### 5.3. Comparison with the OCQ-Max-Fit, OCQ-Greedy and OCQ-Naïve Algorithms

The purpose of the simulation experiments is to verify whether the proposed algorithm can reasonably apply five different types of sensors under complex multi-constraint conditions to achieve the best coverage effect. In order to verify the performance of the algorithm, we compared with the latest compound event algorithms in the next simulations. In this paper, we compare the OCQ-Max-fit, OCQ-Greedy and OCQ-Naïve algorithms with single cost constraints, because the event coverage under multiple constraints has never been studied before in barrier coverage. Experimental results show that the proposed algorithm outperforms the latest algorithms in terms of cost saving, large area barrier coverage and operational efficiency. This section presents the result of our experiments for the compound event barrier coverage technique (ASMP) with respect to OCQ-Max-fit, OCQ-Greedy and OCQ-Naïve, which can be classified into a knapsack constraint and greedy problem. As mentioned before, the ASMP algorithm adopted in our technique is much better than the mechanism of the OCQ-Max-fit, OCQ-Greedy and OCQ-Naïve algorithms.

**Figure 2:**
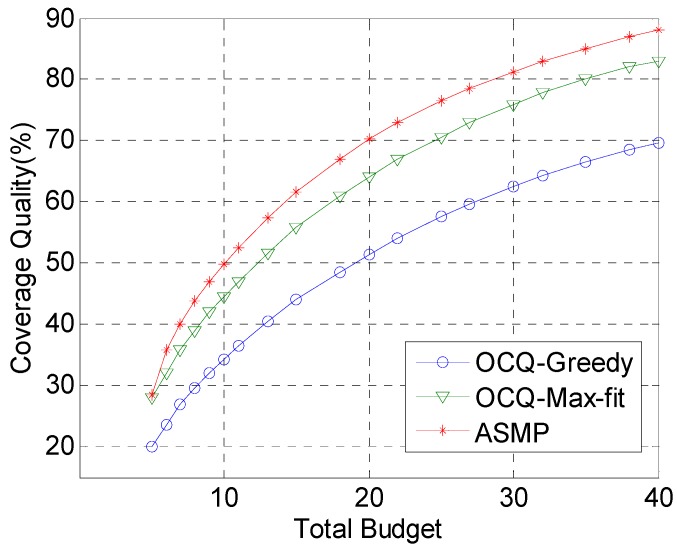
Comparison of the coverage quality when the total budget is set to various values, OCQ-Greedy, OCQ-Max-fit and ASMP are utilized, respectively, in our experiments. This figure shows that the coverage quality increases significantly when the total budget is set to a relatively large value.

Figure 2 shows the coverage quality when the total budget is set to different values for the OCQ-Greedy, OCQ-Max-fit and ASMP algorithm, respectively. With the increase of the budget, the quality of coverage increases. On the other hand, the coverage quality optimized by our technique is much better than that achieved by OCQ-Greedy and OCQ-Max-fit, when the total budget which is restrained by budgetary constraints is relatively more. This is due to the fact that when the total budget is relatively large, the number of sensor nodes and computational complexity increase significantly. The OCQ-Greedy algorithm based on a greedy algorithm only chooses the current optimal solution, which is probably the local optimal solution. OCQ-Max-fit enumerates the skyline points of deployment schemes to compute the coverage quality. Enumeration methods are very simple and only suitable for a relatively small network scale. However, ASMP uses the aggregate function to approximate the maximal function, and only a small part of the functions are involved in the computation. Therefore, the gradient computation is reduced significantly, which reduces the computational cost and is more suitable for large-scale networks.

**Figure 3:**
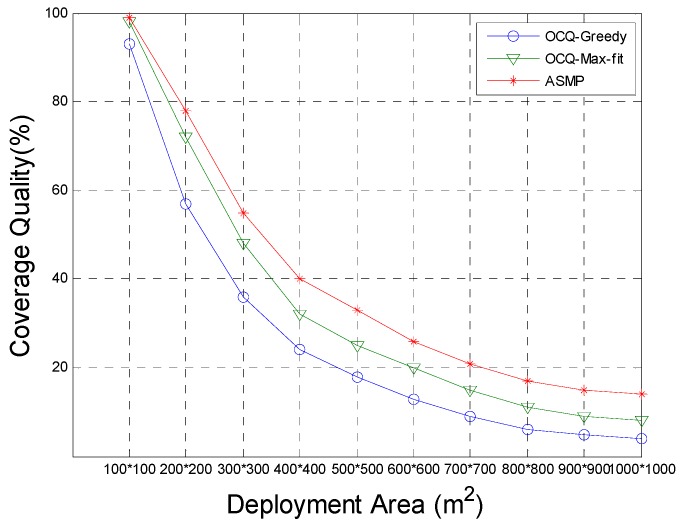
Comparison of the coverage quality for our method (ASMP) with respect to the OCQ-Greedy and OCQ-Max-fit coverage algorithm, when the deployment area A is set to various values, and the coverage quality, which changes with the deployment area A, is set to various values. This figure shows that the coverage quality optimized by our algorithm is much better than that of the OCQ-Greedy and OCQ-Max-fit options, especially when the network deployment area is relatively large.

Figure 3 displays the coverage quality versus deployment area. With the increase of the barrier coverage area, the coverage quality is declining under a limited cost constraint.

The ASMP algorithm can quickly optimize the network performance, so after a large increase in the target area, the target area can still be effectively covered. The coverage quality is significantly better than those achieved with the OCQ-Greedy and OCQ-Max-fit algorithms.

**Figure 4:**
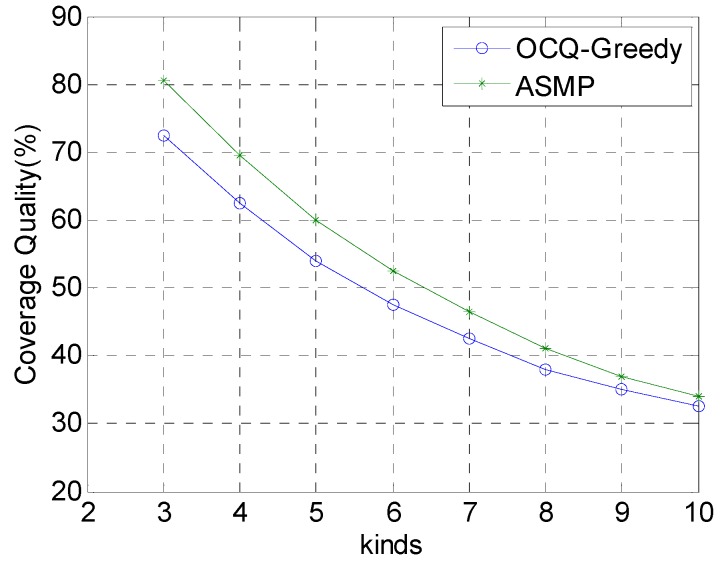
Comparison of the coverage quality for our method (ASMP) with respect to the OCQ-Greedy algorithm when the number of the types of nodes is set to various values, and the coverage quality, which changes with the types of nodes, is set to various values. This figure shows that the coverage quality optimized by our algorithm is much better than that of OCQ-Greedy, especially when the number of the types of nodes is relatively small.

Figure 4 shows the relationship between the coverage quality and the number of the types of nodes. The total confidence of the compound event is distributed equally among each sub-event. The coverage quality becomes worse with the increase of the number of the kinds of the nodes when the total budget is fixed. As the ASMP is more suitable for large-scale complex scenes, when the sensor type increases, its performance is better than that of the OCQ-Greedy algorithm.

**Figure 5:**
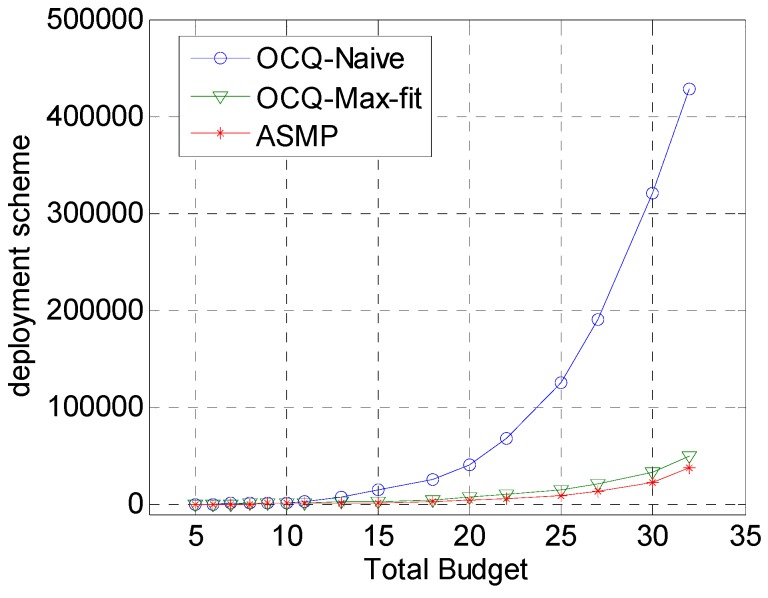
Comparison of deployment schemes for our technique (ASMP) with respect to OCQ-Naïve and OCQ-Max-fit when the total budget is set to various values, respectively, and the deployment schemes, which drift with the total budget, are set to various values. This figure shows that the increase of deployment schemes for our coverage strategy is much slower than that of OCQ-Naïve and OCQ-Max-fit, especially when the total budget is relatively sufficient.

Figure 5 depicts the comparison of the number of deployment schemes derived by OCQ-Naïve, OCQ-Max-fit and ASMP. With the increase of the total budget, each of the algorithms needs to derive more deployment schemes.

However, the growth rate of ASMP is much slower than that of OCQ-Naïve and OCQ-Max-fit, which indicates that ASMP is more efficient than OCQ-Naïve and OCQ-Max-fit. This is because the aggregation function in ASMP can effectively reduce the calculation cost and make the algorithm more efficient.

**Figure 6:**
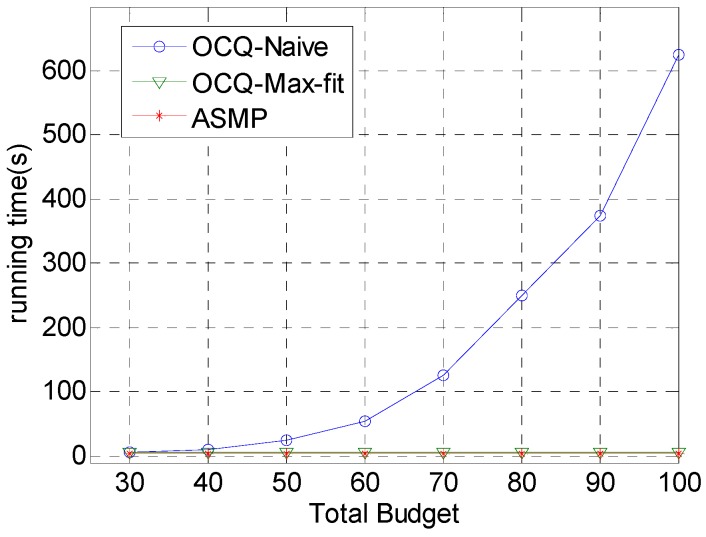
Comparison of the running time for our technique (ASMP) with respect to OCQ-Naïve and OCQ-Max-fit, respectively, when the total budget is set to various values and the running time, which changes with the total budget, is set to various values. This figure shows that the increase of the running time for our coverage strategy is much smaller than that of OCQ-Naïve and a little smaller than that of OCQ-Max-fit, especially when the total budget is relatively sufficient.

Figure 6 demonstrates the comparison of the running time for the three algorithms. The running time of the OCQ-Naïve algorithm grows rapidly with the increase of the total budget. ASMP runs a little faster than OCQ-Max-fit. However, their growth is almost negligible with respect to the OCQ-Naïve algorithm. Compared to OCQ-Naïve and OCQ-Max-fit, ASMP uses the aggregate function to approximate the maximal function, and only a small part of the functions are involved in the computation, therefore, the gradient computation is reduced significantly, which reduces the total computational cost and is more suitable for large-scale networks.

## 6. Conclusions and Future Work

In this paper, the problem of compound event barrier coverage under multiple constraints has been studied. The event model has been composed for sub-events and compound events, and the joint probabilistic method has been used to solve the problem of merging the confidence. Aiming at the problem of multiple constraints in the application of barrier coverage, a multiplier method based on an active-set strategy is proposed. Experimental results show that the proposed algorithm can allocate network resources reasonably well according to the actual situation of the network, effectively improve the coverage quality of the network, and reduce the computation cost of the network. Event coverage with multi-constraint conditions has a wide range of practical applications. In the future, we intend to investigate the barrier coverage in underwater wireless sensor networks based on the actual characteristics of the networks.

## Figures and Tables

**Table 1:** Parameters of Experiments.

Symbol	Meaning	Deployment Time *T*/s	Perceived Radius *R*/m	Cost *C*/s	Confidence S
$\alpha_{1}$	Optical Density Sensor	1	30	25	0.1
$\alpha_{2}$	Temperature Sensor	2	15	10	0.05
$\alpha_{3}$	Video Sensor	5	10	35	0.45
$\alpha_{4}$	Smoke Density Sensor	1	25	20	0.15
$\alpha_{5}$	Infrared Sensor	3	20	15	0.25

**Table 2:** Multi-constraint Parameters under Four Conditions.

Case	Time Constraints	Distance Constraints	Cost Constraints	Minimum Confidences	Coverage Ratio	Running Time
1	47	555	495	0.80	59.7%	<1 s
2	101	1320	1215	0.82	73.6%	<1 s
3	165	1430	1140	0.90	83.5%	<1 s
4	336	1850	2285	0.99	92.5%	<1 s
